# First person – Yong Wang

**DOI:** 10.1242/bio.046714

**Published:** 2019-08-15

## Abstract

First Person is a series of interviews with the first authors of a selection of papers published in Biology Open, helping early-career researchers promote themselves alongside their papers. Yong Wang is first author on ‘Exposure to a 50 Hz magnetic field at 100 µT exerts no DNA damage in cardiomyocytes’, published in BiO. Yong is a PhD student in the lab of Dr Chen at the Huazhong University of Science and Technology, Wuhan, China, investigating cardiovascular pathophysiology.


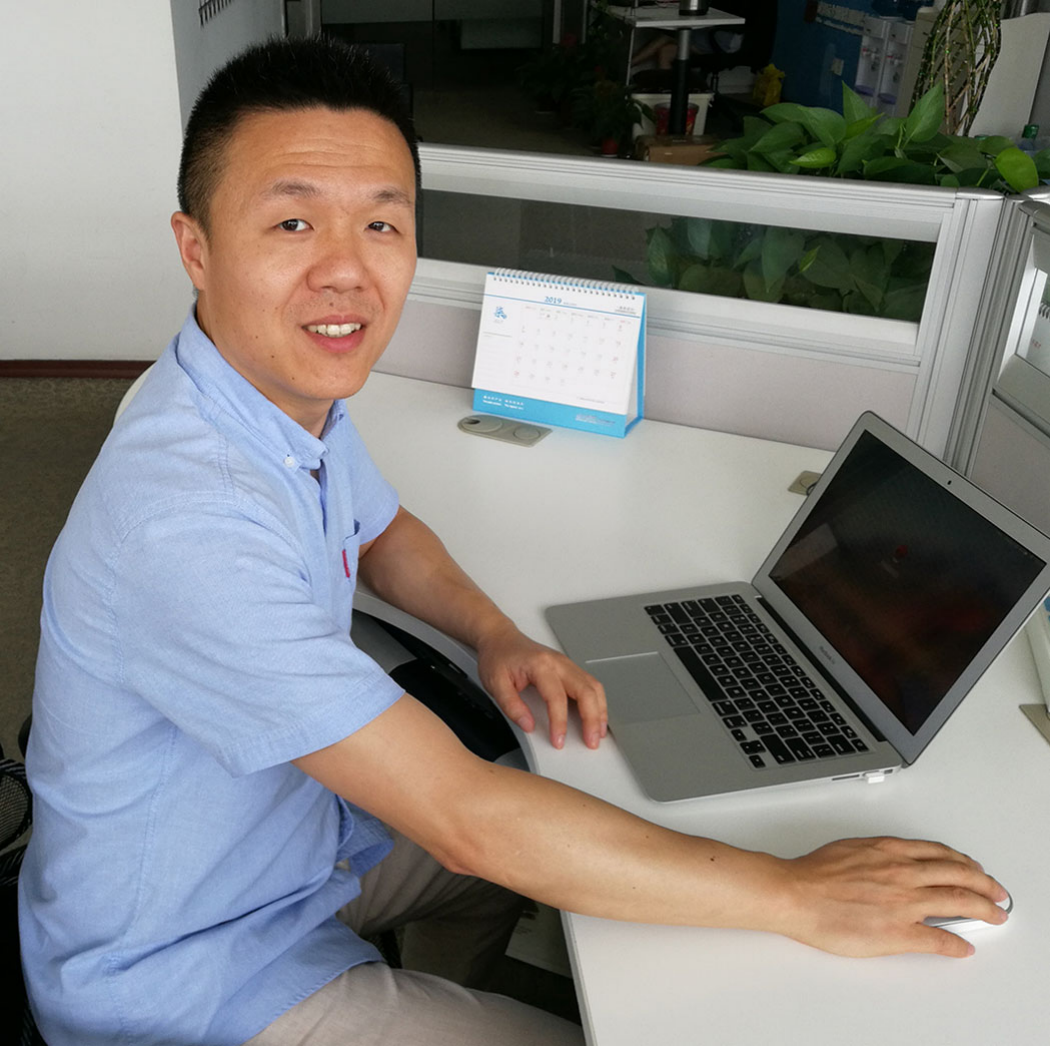


**Yong Wang**

**What is your scientific background and the general focus of your lab?**

I am a PhD student at Huazhong University of Science and Technology in the Division of Cardiology. I am involved in the field of cardiovascular molecular biology, including endothelial cell apoptosis, heart failure and so on.

**How would you explain the main findings of your paper to non-scientific family and friends?**

Electromagnetic fields are generated around transmission lines and appliances due to the presence of a current. Whether these low-frequency and weak-intensity electromagnetic fields have an impact on human health has not been known until now. To this end, many researchers have conducted various research work. We simulated a similar electromagnetic field environment in our experiment and exposed the cardiomyocytes to this condition for a short time to see if the myocardial cell DNA was affected or damaged. The results showed that under such conditions, no evidence was found that the myocardial cell DNA was damaged.

“Our daily lives and electrical appliances are inseparable.”

**What are the potential implications of these results for your field of research?**

The academic community has yet to reach an agreement on the carcinogenicity of low-frequency electromagnetic fields. A solid theoretical foundation or reliable clinical data can help answer this question. However, because of the complexity and particularity of this research field, the above two are difficult to obtain. Like many scholars working in the field, our research provides some new evidence to answer this question. DNA damage is the basis for canceration. In our study, no DNA damage was detected in cardiomyocytes after exposure to low-frequency electromagnetic fields for a short period of time. This indicates that the low-frequency electromagnetic field does not provide the conditions that cause cells to become cancerous.

**What has surprised you the most while conducting your research?**

What surprised me the most in my research was the sensitivity of single-cell gel electrophoresis (also known as the comet assay) to detect DNA damage. I used a different concentration of hydrogen peroxide to treat the cells in the experiment to illustrate the sensitivity of the comet assay protocol I used. According to the literature, the lowest concentration of hydrogen peroxide I used was 2 μM. This concentration is so low that it is at least 20 times lower than the hydrogen peroxide concentration used in some other experiments. Even with such a low concentration of hydrogen peroxide intervention, DNA damage can be detected. It is reported that some laboratories can detect DNA damage caused by 1 μM hydrogen peroxide. Such a highly sensitive detection method provides strong support in explaining that the electromagnetic field does not cause DNA damage.

**What, in your opinion, are some of the greatest achievements in your field and how has this influenced your research?**

There are countless great achievements in the field of biological sciences. From the advent of the DNA double helix structure, the establishment of the rules of genetic information transfer, the discovery of reverse transcription, and the development of gene editing technology, these significant advances are numerous. One of the major achievements I want to mention is the continuous development of biophysics. Biophysics is an interdisciplinary subject combining physics and biology. It is one of the important branches and fields of life science and physics. Biophysics has been integrated into the fields of agriculture, medicine and industry. Studying the physical laws of living matter can not only further clarify the nature of living things, but more importantly, it can make people's understanding of the laws of the whole material movement in the natural world reach new heights.

**Figure:**
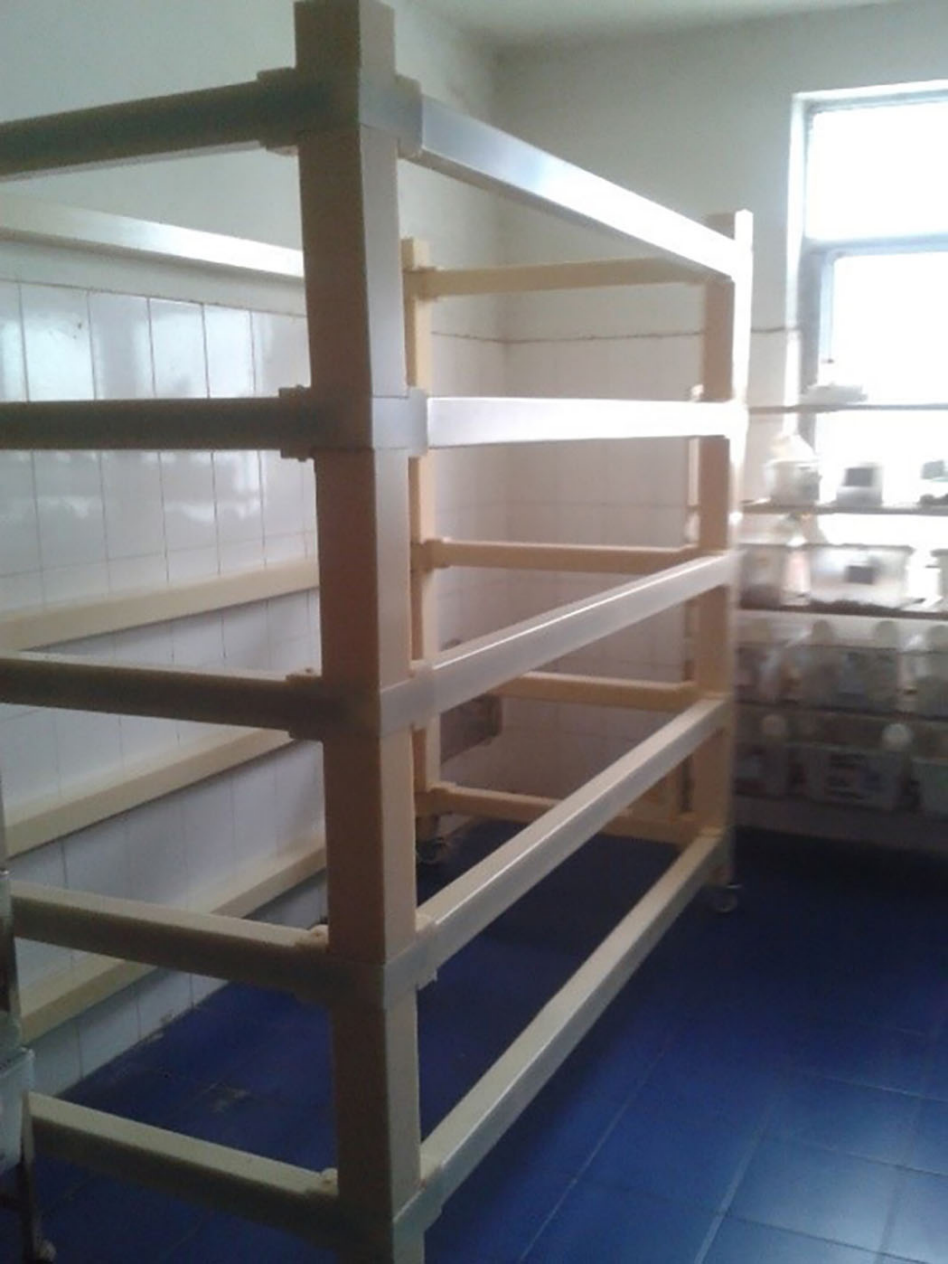
**The exposure device designed and constructed by our team.**

**What changes do you think could improve the professional lives of early-career scientists?**

I think that most of the time researchers are conducting in-depth research in this field. However, the broadening of the professional scope is equally meaningful. This is the case with the electromagnetic field research project I am involved in, involving physics, cell biology and molecular biology. The intersection and integration of disciplines brings greater possibilities for answering such problems. For newcomers to research, not being limited to the individual's professional field, and increasing the involvement of interdisciplinary subjects may be more conducive to the development of the discipline.

“Many unresolved scientific problems are often topics that involve multiple disciplines.”

**What's next for you?**

Our team will continue to conduct research on electromagnetic fields and explore the possible effects of different electromagnetic field exposure conditions on living organisms. I will also be involved. I hope to further study the theoretical basis of the electromagnetic field so that we can design a reasonable test method for exploration. With the help of scientific experimental methods, it is possible to discover the mechanism of the action of electromagnetic fields on organisms.
